# Supplementary material for: A fault-intrusion-tolerant system and deadline-aware algorithm for scheduling scientific workflow in the cloud
Source: PeerJ Comput Sci. 2021 Nov 2;7:e747. doi: 10.7717/peerj-cs.747 (PMC8576554; doi:10.7717/peerj-cs.747)
Supplement: Supplemental Information 6 [file peerj-cs-07-747-s006.zip › Myproject1.1/docs/org/cloudbus/cloudsim/power/PowerDatacenterNonPowerAware.html]

PowerDatacenterNonPowerAware


JavaScript is disabled on your browser.


- Overview
- Package
- Class
- Use
- Tree
- Deprecated
- Index
- Help

- Prev Class
- Next Class

- Frames
- No Frames

- All Classes

- Summary:
- Nested |
- Field |
- Constr |
- Method

- Detail:
- Field |
- Constr |
- Method


org.cloudbus.cloudsim.power

## Class PowerDatacenterNonPowerAware

- java.lang.Object
- - org.cloudbus.cloudsim.core.SimEntity
  - - org.cloudbus.cloudsim.Datacenter
    - - org.cloudbus.cloudsim.power.PowerDatacenter
      - - org.cloudbus.cloudsim.power.PowerDatacenterNonPowerAware

- All Implemented Interfaces:
  :   java.lang.Cloneable

  ---

    

  ```
  public class PowerDatacenterNonPowerAware
  extends PowerDatacenter
  ```

  PowerDatacenterNonPowerAware is a class that represents a non-power aware data center in the
  context of power-aware simulations.
  If you are using any algorithms, policies or workload included in the power package please cite
  the following paper:
  Anton Beloglazov, and Rajkumar Buyya, "Optimal Online Deterministic Algorithms and Adaptive
  Heuristics for Energy and Performance Efficient Dynamic Consolidation of Virtual Machines in
  Cloud Data Centers", Concurrency and Computation: Practice and Experience (CCPE), Volume 24,
  Issue 13, Pages: 1397-1420, John Wiley & Sons, Ltd, New York, USA, 2012

  Since:
  :   CloudSim Toolkit 2.0

- - ### Field Summary

    - ### Fields inherited from class org.cloudbus.cloudsim.core.SimEntity

      `FINISHED, HOLDING, RUNNABLE, WAITING`
  - ### Constructor Summary

    Constructors

    Constructor and Description

    `PowerDatacenterNonPowerAware(java.lang.String name,
    DatacenterCharacteristics characteristics,
    VmAllocationPolicy vmAllocationPolicy,
    java.util.List<Storage> storageList,
    double schedulingInterval)`

    Instantiates a new datacenter.
  - ### Method Summary

    Methods

    | Modifier and Type | Method and Description |
    | --- | --- |
    | `protected void` | `updateCloudletProcessing()` Updates processing of each cloudlet running in this PowerDatacenter. |

    - ### Methods inherited from class org.cloudbus.cloudsim.power.PowerDatacenter

      `getCloudletSubmitted, getMigrationCount, getPower, incrementMigrationCount, isDisableMigrations, isInMigration, processCloudletSubmit, processVmMigrate, setCloudletSubmitted, setDisableMigrations, setMigrationCount, setPower, updateCloudetProcessingWithoutSchedulingFutureEvents, updateCloudetProcessingWithoutSchedulingFutureEventsForce`
    - ### Methods inherited from class org.cloudbus.cloudsim.Datacenter

      `addFile, checkCloudletCompletion, contains, contains, getCharacteristics, getHostList, getLastProcessTime, getRegionalCisName, getSchedulingInterval, getStorageList, getVmAllocationPolicy, getVmList, predictFileTransferTime, processCloudlet, processCloudletCancel, processCloudletMove, processCloudletPause, processCloudletResume, processCloudletStatus, processDataAdd, processDataDelete, processEvent, processOtherEvent, processPingRequest, processVmCreate, processVmDestroy, registerOtherEntity, setCharacteristics, setLastProcessTime, setRegionalCisName, setSchedulingInterval, setStorageList, setVmAllocationPolicy, setVmList, shutdownEntity, startEntity`
    - ### Methods inherited from class org.cloudbus.cloudsim.core.SimEntity

      `cancelEvent, clone, getEventBuffer, getId, getName, getNextEvent, getNextEvent, getState, numEventsWaiting, numEventsWaiting, pause, run, schedule, schedule, schedule, schedule, scheduleFirst, scheduleFirst, scheduleFirst, scheduleFirst, scheduleFirstNow, scheduleFirstNow, scheduleFirstNow, scheduleFirstNow, scheduleNow, scheduleNow, scheduleNow, scheduleNow, selectEvent, send, send, send, send, sendNow, sendNow, sendNow, sendNow, setEventBuffer, setId, setState, waitForEvent`
    - ### Methods inherited from class java.lang.Object

      `equals, finalize, getClass, hashCode, notify, notifyAll, toString, wait, wait, wait`

- - ### Constructor Detail


    - #### PowerDatacenterNonPowerAware

      ```
      public PowerDatacenterNonPowerAware(java.lang.String name,
                                  DatacenterCharacteristics characteristics,
                                  VmAllocationPolicy vmAllocationPolicy,
                                  java.util.List<Storage> storageList,
                                  double schedulingInterval)
                                   throws java.lang.Exception
      ```

      Instantiates a new datacenter.

      Parameters:
      :   `name` - the name
      :   `characteristics` - the res config
      :   `schedulingInterval` - the scheduling interval
      :   `utilizationBound` - the utilization bound
      :   `vmAllocationPolicy` - the vm provisioner
      :   `storageList` - the storage list

      Throws:
      :   `java.lang.Exception` - the exception
  - ### Method Detail


    - #### updateCloudletProcessing

      ```
      protected void updateCloudletProcessing()
      ```

      Updates processing of each cloudlet running in this PowerDatacenter. It is necessary because
      Hosts and VirtualMachines are simple objects, not entities. So, they don't receive events and
      updating cloudlets inside them must be called from the outside.

      **Overrides:**
      :   `updateCloudletProcessing` in class `PowerDatacenter`


- Overview
- Package
- Class
- Use
- Tree
- Deprecated
- Index
- Help

- Prev Class
- Next Class

- Frames
- No Frames

- All Classes

- Summary:
- Nested |
- Field |
- Constr |
- Method

- Detail:
- Field |
- Constr |
- Method
